# Towards sustainable horizons: A comprehensive blueprint for Mars colonization

**DOI:** 10.1016/j.heliyon.2024.e26180

**Published:** 2024-02-15

**Authors:** Florian Neukart

**Affiliations:** a Leiden Institute of Advanced Computer Science, Snellius Gebouw, Niels Bohrweg 1, Leiden, 2333 CA, South Holland, Netherlands; b Terra Quantum AG, Kornhausstrasse 25, St. Gallen, 9000, St. Gallen, Switzerland

**Keywords:** Mars colonization, Radiation shielding, In-situ resource utilization, Solar sail propulsion, Martian infrastructure, Aeroponic food production, Economic modeling, Reduced gravity health effects, Martian communication relay, Algae bioreactors

## Abstract

This paper thoroughly explores the feasibility, challenges, and proposed solutions for establishing a sustainable human colony on Mars. We quantitatively and qualitatively analyze the Martian environment, highlighting key challenges such as radiation exposure, which astronauts could experience at minimum levels of 0.66 sieverts during a round trip, and the complications arising from Mars' thin atmosphere and extreme temperature variations. Technological advancements are examined, including developing Martian concrete, which utilizes sulfur as a binding agent, and innovative life support strategies like aeroponics and algae bioreactors. The human aspect of colonization is addressed, focusing on long-term space habitation's psychological and physiological impacts. We also present a cost-benefit analysis of in-situ resource utilization versus Earth-based supply missions, emphasizing economic viability with the potential reduction in launch costs through reusable rocket technology. A timeline for the colonization process is suggested, spanning preliminary unmanned missions for resource assessment, followed by short-term manned missions leading to sustainable settlements over several decades. The paper concludes with recommendations for future research, particularly in refining resource utilization techniques and advancing health and life support systems, to solidify the foundation for Mars colonization. This comprehensive assessment aims to guide researchers, policymakers, and stakeholders in planning and executing a strategic and informed approach to making Mars colonization a reality.

## Highlights

• Analysis of Mars' environmental and radiation challenges.
• Sustainable infrastructures like Martian concrete and subterranean cities.
• Diverse energy methods for Mars: solar to nuclear.
• Innovative transport: solar sails, specialized rovers.
• Life support focus: water, aeroponics, and bio-reactors.

## 1 Introduction

The quest to transcend our planetary boundaries and establish a presence on another celestial body is one of the most ambitious endeavors undertaken by humanity [1], [2]. The allure of space exploration, which once captivated humanity through mythical narratives and astronomical studies, has evolved into a tangible aspiration in the modern era. This aspiration is fueled by a blend of scientific curiosity, technological prowess, and a profound desire to seek answers to fundamental questions about our existence and the universe. Among the celestial bodies within our reach, Mars, with its relative proximity and some similarities to Earth, emerges as the prime candidate for colonization [3]. With its mysteries and untapped potential, the Red Planet is a new frontier for human exploration and habitation. Establishing a sustainable human presence on Mars is an engineering challenge and a testament to human determination, resilience, and ingenuity. The vision of Mars colonization transcends the mere act of landing humans on another planet; it encompasses the creation of a new domain for human life, replete with its own unique challenges and opportunities [4]. This vision is propelled by an understanding that the future of humanity might not be confined to the cradle of Earth. Mars is a beacon in this journey, offering a canvas for one of the most profound chapters in human exploration. The efforts to reach and settle on Mars could redefine our technological capabilities, expand our scientific knowledge, and perhaps most importantly, cement the idea that our species can thrive beyond the confines of our native planet. The potential colonization of Mars also carries with it a sense of urgency and a safeguard against global threats to humanity. In an age where Earth's vulnerability to natural and human-made disasters is increasingly evident, Mars offers a potential refuge - a ‘Plan B’ for civilization [4]. This notion of planetary backup extends beyond mere survival; it is about ensuring the continuation of human culture, knowledge, and the diverse tapestry of life that our planet harbors. The colonization of Mars could be an insurance policy against catastrophic events that could imperil human existence on Earth, such as asteroid impacts, ecological collapse, or other unforeseeable threats. Furthermore, the journey to Mars is not solely a journey outward but also a voyage of introspection and reflection. It compels us to confront fundamental questions about our place in the universe and our responsibilities as a spacefaring species. The challenges and ethical considerations of establishing a permanent human settlement on another planet prompt us to reevaluate our understanding of stewardship, coexistence, and our long-term aspirations as a species. As we embark on this audacious journey, the quest to colonize Mars stands as a testament to our unyielding spirit and the relentless pursuit of knowledge and adventure that defines us as human beings.

The endeavor to establish a human presence on Mars represents a convergence of numerous scientific, technological, and existential considerations. This ambitious goal, evolving from the realms of early astronomical studies and science fiction into a tangible objective, is now at the forefront of modern space exploration. The allure of Mars, with its similarities to Earth and proximity, makes it an ideal target for colonization, offering a new frontier for human habitation and many scientific opportunities [1], [3].

The challenge of Mars colonization encompasses diverse and complex issues, ranging from technological advancements in space travel to the sustainability of life in a harsh extraterrestrial environment. Previous research has primarily focused on individual aspects of Mars missions, such as propulsion technologies [5], habitat design [6], and life support systems [7]. However, many comprehensive studies integrate these multifaceted challenges into a cohesive approach for sustainable colonization. Recent advancements in space technology, particularly in reusable launch systems [8], and growing interest from private entities like SpaceX and Blue Origin [9], have brought Mars colonization within the realm of possibility. However, current literature often overlooks the interconnected nature of technological, human, environmental, and economic factors that are critical to the success of such a mission [10], [11].

This study addresses the research gap by providing a holistic view of Mars colonization. It integrates various aspects of space exploration that have been traditionally studied in isolation, offering a comprehensive blueprint that considers technological, human, environmental, and economic dimensions. This approach is particularly pertinent given the increasing feasibility of Mars colonization and the need for a sustainable and multidisciplinary strategy [4], [12].

The uniqueness of this study lies in its integrative approach, combining insights from engineering, space science, human factors, environmental ethics, and economics to provide a strategic framework for Mars colonization. Unlike previous works focusing on specific technical or scientific challenges, this study presents a broader perspective, emphasizing the need for a balanced and sustainable approach to colonizing Mars [13], [14]. By highlighting the interdependencies among the various aspects of Mars missions, the study contributes to a more comprehensive understanding of what it would take to establish a sustainable human presence on the Red Planet.

This research is significant as it contributes to the ongoing discourse on space exploration, providing valuable insights for policymakers, scientists, engineers, and space exploration enthusiasts. It underscores the importance of a multidisciplinary approach in tackling one of the most ambitious endeavors of the 21st century — establishing a human colony on Mars [15], [16]. The study advances our understanding of the challenges and opportunities associated with Mars colonization and highlights the broader implications of such an endeavor for human society and our future in space.

### 1.1 Need for colonization

The rationale behind colonizing Mars is multifaceted, driven by a blend of scientific, existential, and strategic motivations [1]:

• **Planetary Backup**: Our planet, Earth, is not immune to a range of existential threats. These include natural disasters like asteroid impacts, which have historically caused mass extinctions, and supervolcano eruptions that could lead to catastrophic climate effects [4]. Additionally, human-made dangers such as nuclear warfare, biotechnology risks, or runaway climate change pose significant threats to our continued existence on Earth [17]. Establishing a human settlement on Mars provides a form of insurance for humanity, a safeguard against the extinction of our species, and a repository for preserving our cultures, knowledge, and biological diversity.
• **Scientific Exploration**: Mars is a scientific treasure trove, presenting an opportunity to expand our knowledge about the solar system. Studying its geology, atmosphere, and potential subsurface water or ice reserves can provide crucial insights into the past habitability of Mars and the potential for life [18]. Understanding Martian climate and geologic history is essential for comparative planetology – comparing and contrasting the evolutionary paths of Earth and Mars. This could reveal vital information about planetary systems in general, including the conditions necessary for life and how planetary environments can change over time [19].
• **Technological Advancement**: The endeavor to colonize Mars presents complex engineering and technological challenges, from developing spacecraft capable of transporting humans to Mars to designing habitats that can sustain life in a harsh environment. Addressing these challenges necessitates innovation in various fields, including propulsion systems, life support, sustainable energy solutions, and advanced materials. These technological breakthroughs have the potential for significant terrestrial applications, such as advancements in renewable energy technologies, efficient recycling systems, and robotics. They may also catalyze further explorations and expeditions to other celestial bodies [5], [10].
• **Inspiration and Education**: Pursuing Mars colonization is a powerful source of inspiration. It captures human imagination, embodying the spirit of exploration and the desire to push the boundaries of our capabilities. This endeavor can inspire new generations of scientists, engineers, and space enthusiasts. It can galvanize public interest in science, technology, engineering, and mathematics (STEM) fields, increasing educational opportunities and fostering a culture of innovation and discovery [20].
• **Economic Opportunities**: Beyond the scientific and survival imperatives, Mars colonization could open new economic frontiers. This includes the potential extraction and utilization of Martian resources, ranging from mining minerals and metals to utilizing Martian regolith for construction. Developing Martian infrastructure and habitats could also pave the way for future space tourism, offering unique travel experiences and economic opportunities [12], [16].
• **International Collaboration and Peace**: The endeavor to colonize Mars requires a level of international cooperation and collaboration unprecedented in human history. It allows nations to unite, pooling resources, knowledge, and expertise towards a common goal. Such a global effort can act as a unifying force, promoting international peace and collaboration and setting a precedent for how humanity can work together to solve grand challenges [9], [15].

The multifaceted rationale for colonizing Mars underscores its significance as a milestone in human evolution. It represents an amalgamation of survival strategy, scientific inquiry, technological prowess, economic potential, cultural inspiration, and international cooperation. As we set our sights on this red neighbor in our solar system, Mars colonization emerges not just as a feasible venture but as a necessary step in the journey of human progress and survival.

### 1.2 Current challenges

While the allure of Mars is compelling, the challenges of establishing a sustainable human presence on the Red Planet are formidable and multifaceted [10]:

• **Vast Distance from Earth**: The immense distance between Earth and Mars presents significant logistical and communication challenges. Resupply missions to Mars would be infrequent, take months to reach the planet, and require precise planning and significant resources [21]. Communication with Earth would also be subject to non-negligible latency, ranging from 4 to 24 minutes one way. This delay impacts personal communication and mission control operations, requiring a high degree of autonomy for Mars settlers [22].
• **Thin Atmosphere and Lack of Magnetosphere**: Mars' thin atmosphere, composed primarily of carbon dioxide, provides minimal protection from harmful cosmic and solar radiation [23]. The absence of a global magnetosphere further exposes the surface to high radiation levels, posing significant risks to human health, including increased cancer risks, radiation sickness, and potential genetic damage [24]. This necessitates the development of advanced radiation shielding for habitats, spacesuits, and any surface activities.
• **Extreme Temperatures and Reduced Atmospheric Pressure**: The Martian environment is characterized by extreme temperatures, with daily fluctuations ranging from relatively mild temperatures to well below freezing [5]. The reduced atmospheric pressure, less than 1% of Earth's at sea level, makes it impossible for humans to survive without pressurized habitats and spacesuits. These conditions require robust and reliable life support systems that can regulate temperature and pressure, manage oxygen levels, and ensure the sustainability of human life in such an alien environment [6].
• **Reduced Gravity**: Mars has a gravitational force about 38% that of Earth's. While this reduced gravity allows for easier movement and less energy expenditure, it may have adverse long-term effects on human health [7]. Prolonged exposure to lower gravity levels can lead to muscle atrophy, bone density loss, and changes in cardiovascular and other bodily systems. Understanding and mitigating these health impacts is crucial for the long-term sustainability of human life on Mars [25].
• **Psychological Challenges**: The isolation, confinement, and remote nature of a Mars settlement pose significant psychological challenges for settlers. These include dealing with the monotony of the environment, managing interpersonal dynamics in a small group, and coping with the knowledge of being millions of kilometers away from Earth [26]. Addressing these challenges requires careful selection of crew members, extensive training in psychological resilience, and the development of support systems and activities to maintain mental health and morale [27].
• **Environmental and Planetary Protection Concerns**: Colonizing Mars raises important environmental and ethical considerations. These include the potential contamination of Mars with Earth microbes, which could jeopardize the search for indigenous Martian life and alter the Martian ecosystem [14]. Additionally, ethical questions about the human alteration of another planetary body must be considered, balancing the scientific and exploration goals with the need to preserve Mars's natural state [13].

These challenges highlight the complexity of establishing a human presence on Mars. Addressing them requires technological innovation, robust engineering solutions, and considerations of human health, psychology, environmental impact, and ethical implications. The successful colonization of Mars depends on a multidisciplinary approach that integrates science, technology, human factors, and ethical considerations into a cohesive and sustainable strategy.

### 1.3 Significance of sustainable and cost-effective methods

The realization of Martian colonization hinges on the technological and scientific capability to reach and survive on Mars and the sustainability and economic feasibility of such missions [11]. The traditional models of space exploration, heavily reliant on Earth for supplies and support, are impractical for the scale and duration of Mars colonization efforts. Therefore, developing sustainable and cost-effective methods for living and working on Mars is crucial:

• **Economic Feasibility**: The cost of transporting even the most essential supplies from Earth to Mars is astronomically high, given the distances and energy requirements for interplanetary travel [22]. This necessitates a paradigm shift towards using Martian resources (in-situ resource utilization, or ISRU) to support human life, including water extraction, oxygen production, and building materials. This approach reduces the launch mass and cost and enhances the mission's autonomy and resilience [28], [29].
• **Reduced Dependence on Earth**: Given the communication delay of up to 24 minutes one way and the potential for periods of no communication, reliance on Earth for decision-making, problem-solving, and resupply is infeasible [7]. Achieving high self-sufficiency in energy production, food cultivation, and manufacturing is essential. This includes developing renewable energy systems like solar and nuclear power, closed-loop life support systems, and 3D printing technologies for tool and component manufacturing [30], [31].
• **Environmental and Ethical Responsibility**: Mars's exploration and potential settlement must be conducted to minimize the impact on the Martian environment. This involves preventing biological contamination, preserving Martian sites of scientific interest, and ensuring that activities on Mars do not irreversibly alter the planet's environment [13], [14]. Ethical considerations in utilizing Martian resources and altering the Martian landscape also come into play, requiring thoughtful stewardship and international cooperation [32].
• **Technological Innovation and Scalability**: The development of sustainable technologies for Mars colonization has broader implications for Earth. Innovations in energy efficiency, waste recycling, and habitat construction on Mars can inform sustainable practices and technologies on Earth. Furthermore, these technologies must be scalable and adaptable to support a growing and evolving Martian settlement [15], [16].
• **Socio-Economic Dynamics and Public Engagement**: The economic models of Mars colonization, including public-private partnerships and the role of international collaborations, significantly impact the mission's structure and governance. Public engagement and support are vital for these missions' long-term success and sustainability, necessitating transparent communication and outreach efforts [1], [9].

### 1.4 Historical and technological progression toward Mars colonization

*Context and historical perspective:*  The journey to Mars colonization began with early astronomical observations and expanded through scientific and engineering advancements. Fueled by science fiction, initial fascination transformed as space exploration became tangible with missions like the Viking programs and the Curiosity rover. These expeditions provided invaluable insights into Martian geology, atmosphere, and potential habitability, marking a transition from Mars as a distant enigma to a feasible target for human exploration [33], [34].

*Technological evolution:*  The evolution of space technology is central to the feasibility of colonization on Mars. Key advancements include developing efficient propulsion systems, robust habitat designs, sustainable life support systems, and advanced robotics. Notably, as spearheaded by companies like SpaceX, the advent of reusable rocket technology offers a potential reduction in the immense costs associated with interplanetary travel. Concurrently, the rise of 3D printing technology holds promise for constructing habitats directly from Martian materials, a critical component of in-situ resource utilization [8], [35], [36], [37].

### 1.5 Challenges and considerations in Martian colonization

*Scientific discoveries and environmental challenges:*  The wealth of data from Mars exploration missions has reshaped our understanding of the Red Planet. Discoveries of past water flows and essential life-sustaining elements underscore Mars's potential past habitability. However, settling on Mars introduces unique environmental and health challenges, notably the increased exposure to cosmic and solar radiation due to the thin Martian atmosphere and lack of a global magnetic field. These challenges extend to the human body, with research ongoing into the effects of prolonged exposure to Mars's reduced gravity [7], [23], [24], [38], [39], [40].

*Socio-political, ethical, and economic considerations:*  The ambition to colonize Mars extends beyond technological and scientific realms, raising significant socio-political, ethical, and economic questions. These include concerns around planetary protection, the ethical implications of extraterrestrial resource appropriation, and the potential environmental impacts on Mars. The economic aspect, driven by both governmental and private sector interests, has become increasingly prominent. Companies like SpaceX and Blue Origin are pivotal in advancing Mars missions' economic feasibility. Moreover, Mars colonization holds potential as a platform for future space exploration, serving as a springboard for missions beyond the Martian orbit [8], [9], [13], [14], [41], [42].

*Inspiring the future:*  The vision of Mars colonization continues to inspire and educate, capturing public interest and motivating the next generation of explorers. Through educational initiatives and public engagement, Mars missions are fostering a deeper understanding of the importance and impact of space exploration. As efforts and collaborations continue to advance, the prospect of human life thriving on Mars transforms from a distant dream into an approaching reality [15], [16], [43], [44].

## 2 Background

Mars, often dubbed the ‘Red Planet’, has been an object of human curiosity and scientific investigation for centuries. With advances in space technology over the latter half of the 20th century and into the 21st century, our understanding of Mars has transitioned from telescopic observations to detailed in-situ investigations by robotic emissaries [18], [45].

### 2.1 Early observations and speculations

Mars has been known since ancient times due to its visible reddish appearance in the night sky. The planet's distinctive color is due to iron oxide (rust) on its surface [46]. This observation, easily visible to the naked eye, has historically led to various interpretations and mythologies surrounding Mars across different cultures. The scientific study of Mars took a significant turn in the late 19th century with the advent of more advanced telescopic technologies. Astronomers such as *Giovanni Schiaparelli* embarked on detailed observations of the Martian surface. Schiaparelli's mapping of Mars in 1877 and his description of ‘canali,’ an Italian term misconstrued as ‘canals,’ sparked widespread interest and speculation [47]. These observations suggested the presence of long, straight lines on the Martian surface, fueling theories about their artificial construction. The idea of Martian canals was significantly boosted by American astronomer *Percival Lowell*. Lowell was deeply influenced by Schiaparelli's findings and conducted his observations, dedicating much of his career to studying Mars. Between 1894 and 1908, he published several books and delivered numerous lectures where he argued fervently for the existence of canals on Mars, which he believed were evidence of an intelligent Martian civilization [48]. Lowell's theories were based on extensive, albeit subjective, observations made using his observatory in Flagstaff, Arizona. Lowell's work on Martian canals was both influential and controversial, igniting public interest and scientific debate. While his theories were later disproven, they were crucial in promoting interest in Martian studies. Lowell's insistence on the existence of Martian canals led to increased funding and interest in astronomical research, indirectly aiding the advancement of the field. The early 20th century saw a gradual shift in the scientific community's view of Mars. As observational techniques improved and our understanding of the solar system deepened, the idea of Martian canals and an inhabited Mars began to wane. The development of spectroscopy, for instance, allowed astronomers to analyze the Martian atmosphere and surface composition more accurately. These studies revealed a lack of substantial water and a thin atmosphere, conditions unsuitable for the kind of life forms that Lowell had proposed [47], [49]. Additionally, the advent of space probes in the mid-20th century further transformed our understanding of Mars. The Mariner missions of the 1960s provided the first close-up images of the Martian surface, which showed no signs of the canals that Lowell had famously hypothesized [50]. These missions also began to unravel the complexities of Martian geology and atmosphere, laying the groundwork for future exploration. Despite the discrediting of Lowell's theories, his influence persisted. The fascination he helped foster led to Mars being a central focus in early science fiction literature, often depicting the planet as a world teeming with life and civilizations. Authors such as H.G. Wells and Edgar Rice Burroughs were inspired by the idea of Martian canals, weaving tales that captured the public's imagination and kept Mars at the forefront of extraterrestrial exploration in popular culture [51], [52]. In retrospect, the early observations and speculations about Mars illustrate the evolving nature of scientific inquiry and the impact of cultural and technological contexts on our understanding of the cosmos. These initial steps in observing and interpreting Mars not only enriched our knowledge of astronomy but also spurred public interest and scientific momentum that continues to drive Martian exploration to this day.

### 2.2 Robotic exploration

Robotic emissaries have largely carried out the journey to understand and explore Mars. From early reconnaissance missions to advanced rovers and orbiters, robotic exploration has been pivotal in unraveling the mysteries of the Red Planet. This journey of discovery has transformed our understanding of Mars, laying the groundwork for future human exploration. In this section, we trace the progression of these missions, highlighting their contributions and the evolving technologies that made them possible.

#### 2.2.1 Early probes and flybys

The space age initiated the era of robotic Mars exploration. The first attempts by the Soviet Union's *Mars* program in the early 1960s faced challenges, with several missions failing either during launch, en-route, or upon arrival [53]. These early attempts paved the way for more advanced and reliable exploration technologies.

The USA's *Mariner* program was the first to successfully send back data from Mars [54]. *Mariner 4*, in 1965, performed the first successful flyby, revealing a barren landscape, contrary to previous speculations of a wetter Mars. These images provided the first close-up photographs of another planet, altering our perception of Mars from a potentially Earth-like world to a more barren and alien environment.

#### 2.2.2 Landers and the quest for life

The 1970s saw the ambitious *Viking* program by NASA, aiming to land on and study the Martian surface directly. *Viking 1* and *Viking 2* landers undertook experiments to detect microbial life. Although the results from the labeled release experiment initially indicated possible metabolic activity, subsequent analysis suggested that the reactions were more likely due to inorganic processes [55], [56]. The Viking missions marked a significant leap in our ability to conduct complex experiments remotely on another planet.

#### 2.2.3 Rovers: mobile geology labs

In the late 1990s and into the 2000s, rovers became central to Mars exploration. *Pathfinder's* Sojourner was the first, followed by the twin rovers *Spirit* and *Opportunity*. These missions revolutionized our understanding of Martian geology and climate history [50]. Notably, *Opportunity* discovered evidence of ancient liquid water flows on the Martian surface [57]. The rovers' capability to traverse and analyze the Martian terrain brought a new dimension to the exploration, enabling detailed geological studies across varied locations.

The car-sized *Curiosity* rover, part of NASA's *Mars Science Laboratory* mission, landed in 2012. Equipped with a suite of sophisticated instruments, *Curiosity* identified an ancient lakebed and detected organic molecules, further intensifying the search for potential past life [19], [58]. This advanced rover has been able to conduct more comprehensive scientific research, providing invaluable data on Mars' habitability.

#### 2.2.4 Orbiters: eyes in the Martian sky

Complementing the surface missions, a series of orbiters have provided crucial data about Mars' atmosphere, climate, and geology. Notable missions include NASA's *Mars Reconnaissance Orbiter* (MRO) and ESA's *Mars Express* [59], [60]. MRO's HiRISE camera has captured high-resolution images, aiding in landing site selection and studying geological features, while its SHARAD instrument has probed subsurface layers. These orbiters have been instrumental in mapping Mars' surface, studying its climate and weather patterns, and providing critical communication relays for surface missions.

### 2.3 Recent endeavors and international collaboration

The 2020s saw a global convergence towards Mars exploration. NASA's *Perseverance* rover and its innovative *Ingenuity* helicopter, the UAE's *Hope* orbiter, and China's *Tianwen-1* mission, which includes both an orbiter and a rover, all reached Mars in 2021 [61], [62]. These missions represent a new era of exploration, characterized by international cooperation and advanced technology. Collaborative efforts, both international and between governmental and private entities, are seen as the pathway forward.

### 2.4 Prelude to human exploration

With the wealth of data from robotic missions, plans for human exploration and eventual colonization have gained momentum. Both NASA and ESA have outlined potential manned missions to Mars in their future roadmaps, viewing the Moon as a testing ground. Private entities, particularly SpaceX, envision large-scale colonization efforts [63], [64].

While the allure of Mars colonization is compelling, carefully synthesizing our learnings from robotic missions, rigorous research into life support and habitat systems, and international collaboration are vital to turn this vision into a reality [65].

## 3 Martian environment and challenges

The Martian environment presents many unique challenges that must be addressed to facilitate human exploration and potential settlement. These multidimensional challenges range from extreme climatic conditions to technological and health-related issues. This section explores these various challenges, focusing primarily on one of the most significant concerns: radiation exposure.

### 3.1 Radiation

One of the most pressing challenges for human exploration and potential colonization of Mars is the intense radiation from galactic cosmic rays (GCRs) and solar energetic particles (SEPs) [24]. Unlike Earth, Mars lacks a strong magnetosphere and a thick atmosphere, both of which significantly attenuate the incoming cosmic and solar radiation on our home planet.

#### 3.1.1 Sources of radiation

Two primary sources of space radiation would affect Mars explorers:

• **Galactic Cosmic Rays (GCRs):** These are high-energy protons and heavy ions originating from sources outside our solar system, including supernovae. Their energy levels are so high that they can penetrate even the most advanced shielding technologies currently available [66].
• **Solar Energetic Particles (SEPs):** These are sporadic bursts of radiation primarily composed of protons, emitted from the Sun during solar flares and coronal mass ejections [67].

#### 3.1.2 Effects on human health

Prolonged exposure to space radiation has several potential health effects [68]:

• Increased risk of cancer.
• Acute radiation sickness from intense radiation bursts, such as significant solar flares.
• Degenerative tissue effects, including cataract formation, cardiovascular diseases, and potential central nervous system damage.
• Possible infertility and hereditary effects.

#### 3.1.3 Quantifying Martian radiation

Measurements by the *Radiation Assessment Detector (RAD)* on the *Curiosity* rover revealed that an astronaut would be exposed to a minimum of 0.66 sieverts during a round trip to Mars, with a stay on the surface [24]. This is more than three times the radiation limit recommended for astronauts during their entire career.

Given Eq. (1):

(1) $$D=\int_{0}^{T}R(t)dt$$

where *D* is the cumulative radiation dose in sieverts (Sv), R(t) is the radiation rate as a function of time, and *T* is the total time of exposure, we can integrate over the duration of the Mars mission to determine the total dose an astronaut would receive.

#### 3.1.4 Mitigating radiation exposure

Several strategies are under consideration to mitigate the radiation risk:

• **Advanced Shielding:** Using materials such as water, polyethylene, or novel materials like hydrogen-rich boron nitride nanotubes for spacecraft and habitats [69], [70].
• **Underground Habitats:** Constructing habitats below the Martian surface can use Mars' regolith as a natural radiation barrier [71].
• **Magnetic Shields:** Generating a protective magnetosphere around the habitat or spacecraft requires significant energy [23], [70].
• **Pharmaceutical Countermeasures:** Drugs that can mitigate radiation damage or boost the body's natural repair mechanisms [72].

Conclusively, while radiation is a significant challenge for Mars exploration and colonization, ongoing research aims to develop viable solutions to ensure the safety and health of future Martian explorers and settlers [71].

### 3.2 Dust storms

Mars is infamous for its planet-wide dust storms, which can last for weeks to months, enveloping the entire surface in a thick haze [43]. While these storms are not accompanied by the high winds seen in terrestrial storms due to the thin Martian atmosphere [73], they pose unique challenges for human exploration and colonization. Understanding and mitigating the effects of these dust storms is crucial for the success of both robotic and human missions to Mars.

#### 3.2.1 Characteristics of Martian dust storms

• **Frequency:** Localized dust storms frequently occur on Mars, but approximately once every three Martian years (or roughly 5.5 Earth years), the conditions allow these local storms to expand and merge, covering the entire planet [43].
• **Composition:** Martian dust particles are extremely fine, comparable to talcum powder, and are composed mainly of iron oxides, giving Mars its characteristic red color [74].
• **Electrostatic Nature:** Due to the continuous bombardment by ultraviolet (UV) radiation, these dust particles can become electrostatically charged, causing them to cling to surfaces [75].

#### 3.2.2 Challenges posed by dust storms

• **Solar Power Generation:** One of the immediate challenges of dust storms is the significant reduction in sunlight, which can drop to less than 1% of normal levels during a global dust storm [76]. This poses a threat to missions that rely on solar power. For instance, the Opportunity rover's mission was terminated after it lost power during a massive dust storm in 2018 [77].
• **Thermal Management:** Dust accumulation on equipment and habitats can disrupt thermal control by insulating equipment or blocking radiators, leading to overheating [78].
• **Equipment Wear and Tear:** Martian dust's fine, abrasive nature can lead to wear and tear on equipment, especially moving parts like joints and wheels on rovers or seals on habitats [74].
• **Human Health:** The dust's fine nature poses a potential respiratory hazard for astronauts. If it's tracked inside habitats, it could be inhaled and cause health issues [79]. Additionally, the iron oxides in the dust might pose chemical risks if ingested or inhaled in significant amounts [80].
• **Visibility and Navigation:** Dust storms can hamper visibility, making navigation and scientific observations challenging [43].

#### 3.2.3 Mitigation strategies

• **Alternative Power Sources:** Exploratory missions and habitats can integrate backup power sources, such as nuclear power or advanced battery storage, to remain operational during prolonged dust storms [76], [80].
• **Protective Coatings:** Equipment can be coated with materials that repel electrostatically charged dust, reducing accumulation [75].
• **Airlocks and Filtration:** Advanced airlock systems and filtration units can reduce the amount of dust brought into Martian habitats, protecting both equipment and astronauts [79].
• **Dust Monitoring and Forecasting:** Integrating meteorological equipment to monitor and forecast dust movements can allow missions to take preventive measures before storms hit their location [78].

While Martian dust storms do not pose an immediate life-threatening danger, as Hollywood might suggest, they do present substantial operational challenges [76], [77]. Future Martian missions must be equipped to handle these challenges to ensure the safety and success of human exploration and colonization efforts. Comprehensive understanding and proactive planning are essential to mitigate the risks posed by these dust storms, ensuring the reliability and longevity of equipment and safeguarding the health of astronauts on the Martian surface. The unique nature of these storms also offers valuable opportunities for scientific study, contributing to our knowledge of Martian meteorology and climate patterns.

### 3.3 Temperature variations

Mars exhibits extreme temperature fluctuations, much more pronounced than those experienced on Earth [81], [82]. This vast temperature range and the thin atmosphere directly impact human habitation and technological equipment functioning on the Martian surface. Understanding and effectively managing these temperature variations is critical for designing habitats, spacesuits, and all equipment intended for Mars.

#### 3.3.1 Characteristics of Martian temperature

• **Daily Fluctuations:** Mars experiences significant diurnal temperature fluctuations, with temperatures at the equator ranging from a maximum of about 20 °C (70 °F) at noon to a minimum of -73 °C (-100 °F) at night [83].
• **Seasonal Variations:** Owing to its axial tilt of 25.2 degrees, almost similar to Earth's 23.5 degrees, Mars undergoes seasons [82]. However, the lengths of the Martian seasons are about twice as long, given its 687-day year. Winters, especially in the polar regions, can see temperatures drop to -125 °C (-195 °F) [81].
• **Atmospheric Influence:** Mars' thin atmosphere, composed mainly of carbon dioxide, does not trap heat effectively [84]. This lack of a substantial greenhouse effect is a key reason for the planet's cold temperatures.

#### 3.3.2 Challenges arising from temperature variations

• **Human Survival:** The extremely cold temperatures can be fatal to humans without appropriate protection [85].
• **Equipment Functionality:** Electronic and mechanical equipment can malfunction or fail when exposed to such low temperatures [86]. Lubricants can freeze, metals can become brittle, and batteries can lose charge rapidly.
• **Thermal Expansion and Contraction:** The significant temperature fluctuations can cause materials to continuously expand and contract, leading to material fatigue and potential structural failures over time [87].
• **Energy Demands:** Maintaining a habitable temperature inside Martian bases would require a consistent and substantial energy source, especially during the long, cold nights and winters [88].

#### 3.3.3 Mitigation strategies

• **Insulated Habitats:** Designing habitats with advanced insulating materials can help maintain a stable internal temperature [6].
• **Subsurface Habitats:** Building habitats below the Martian surface can leverage the ground's natural insulation properties, leading to more stable internal temperatures [89].
• **Thermal Blankets and Heaters for Equipment:** Protecting equipment with thermal blankets and integrating heaters can prevent damage from cold temperatures [86].
• **Utilizing Local Resources:** Ice deposits on Mars can be melted and used in heat exchange systems to help regulate temperatures inside habitats [88].

Adapting to the extreme and fluctuating temperatures of Mars is paramount for the success of future manned missions and potential colonization efforts [6]. Advanced technologies and innovative solutions will be essential to ensure astronauts' safety and equipment's durability in such a challenging environment. The design of habitats and vehicles must consider the Martian environment's thermal stresses. Research into materials and construction methods that can withstand these fluctuations is ongoing and will be critical to ensuring the longevity and reliability of structures and machinery on Mars. Additionally, understanding the Martian climate and weather patterns will aid in the planning and operating missions, ensuring that astronauts can safely navigate and perform tasks on the Martian surface.

### 3.4 Low atmospheric pressure

The atmospheric pressure on Mars is significantly lower than Earth's, averaging around 600 pascals (0.087 psi), which is less than 1% of Earth's mean sea level pressure [90], [91]. This meager atmospheric pressure, primarily composed of carbon dioxide ($CO_{2}$), presents unique challenges for human missions and colonization [1]. Understanding these challenges and developing strategies to mitigate them is crucial for the feasibility of long-term human presence on Mars.

#### 3.4.1 Characteristics of Martian atmospheric pressure

• **Composition:** Mars' atmosphere is about 95% $CO_{2}$, with traces of nitrogen and argon [92]. The absence of a substantial oxygen component starkly contrasts Earth's oxygen-rich atmosphere.
• **Altitude Variations:** The atmospheric pressure on Mars varies with altitude, with the highest pressures (up to 1,155 pascals) found in the depths of the Hellas Basin and the lowest pressures at the top of Olympus Mons [93].
• **Seasonal Variations:** Martian atmospheric pressure exhibits slight seasonal variations due to the sublimation and deposition of $CO_{2}$ from and onto the polar ice caps [94].

#### 3.4.2 Challenges arising from low atmospheric pressure

• **Human Exposure:** Direct exposure to Mars' low-pressure atmosphere would be lethal to humans [95]. In such conditions, bodily fluids would vaporize, leading to a condition called ebullism.
• **EVA Suit Design:** Designing space suits for extended extravehicular activity (EVA) on Mars is challenging due to balancing mobility and maintaining a higher internal pressure [96].
• **Aerodynamic Behavior:** The thin atmosphere affects the aerodynamics of vehicles, making landing and takeoff operations tricky. Traditional parachutes become less effective, demanding alternative or supplemental landing technologies [97].
• **Radiation:** The thin atmosphere provides limited shielding from harmful solar and cosmic radiation [23].

#### 3.4.3 Mitigation strategies

• **Pressurized Habitats:** Designing habitats that can withstand the internal-external pressure differential is crucial [18]. These habitats would maintain an Earth-like atmosphere inside to support human life.
• **Advanced EVA Suits:** New EVA suit designs could utilize mechanical counterpressure to apply direct pressure to the skin, enhancing mobility while maintaining safety [30].
• **Innovative Landing Systems:** Incorporating retro-rockets, inflatable heat shields, or sky cranes can assist in safe landings in the thin Martian atmosphere [98].
• **Local Resource Utilization:** Extracting $O_{2}$ from local resources, such as water ice or the carbon dioxide atmosphere itself (via electrolysis or chemical processes), can help sustain human colonies and refuel rockets [99].

Mars's uniquely low atmospheric pressure necessitates innovative engineering and biomedical solutions [10]. Addressing these challenges is fundamental for ensuring the safety and success of future Martian exploratory and colonization efforts. Developing advanced EVA suits and habitat systems that can adapt to the low-pressure environment is critical. Additionally, adapting existing technology and developing new methods for landing, resource utilization, and environmental control are essential for human survival and activity on Mars. The Martian atmosphere's unique characteristics also open up opportunities for scientific research, which can further our understanding of planetary atmospheres and lead to advancements in atmospheric science and technology.

## 4 Technological solutions

As humanity contemplates the formidable task of establishing a presence on Mars, it becomes clear that existing technologies—while groundbreaking—are not sufficient. Adapting to Mars' unique challenges mandates fresh technological innovations, particularly those that leverage Mars' own resources and the unique aspects of its environment [10], [18]. This section describes the field of Martian-specific technology solutions, spanning infrastructure, resource utilization, transport, and life support.

### 4.1 Infrastructure & habitat

One of the foremost challenges of Martian colonization lies in creating structures that shelter astronauts from the planet's harsh environment and promote sustained human habitation. Habitats on Mars must be robust against radiation, temperature fluctuations, and low atmospheric pressures, all while being feasible to construct with limited resources transported from Earth [23]. Hence, the ideal approach would lean heavily on in-situ resource utilization, transforming Martian raw materials into sturdy, long-lasting habitats [99]. This subsection explores the latest advancements and proposals, examining how we might construct our first homes on another planet.

#### 4.1.1 Martian concrete

For sustainable human colonization on Mars, it is pivotal to utilize local resources, minimizing the need for resource-intensive shipments from Earth [99]. An essential development in this realm is the prospect of Martian concrete using native regolith and Martian-abundant materials [100].

##### Characteristics of Martian regolith

• **Composition:** Martian regolith comprises finely milled rock, soil, and dust [18]. Its iron oxide content imparts the planet's distinctive red color. This regolith spans a spectrum of particle sizes, from fine dust to small rocks.
• **Basaltic Nature:** A predominant component of Martian soil is weathered basalt, a volcanic rock common to the planet [18].
• **Water Content:** Recent revelations indicate that bound water exists in Mars' regolith, albeit in trace amounts [18].

##### Formulation of Martian concrete

• **Binding Agent:** One innovative proposal involves using sulfur, abundant on Mars, as a binding agent. After heating, sulfur transitions to a liquid state and solidifies upon cooling, effectively binding the regolith [100].
• **Strength and Durability:** Preliminary Earth-based trials with Martian regolith simulants and sulfur have demonstrated a compressive strength on par with terrestrial concrete [100]. It also encourages resistance to Martian-specific challenges like radiation and temperature swings.

##### Advantages of Martian concrete

• **Resource Efficiency:** Harnessing local resources dramatically curtails the necessity of importing massive construction materials from Earth, leading to logistical and economic efficiencies [101].
• **Quick Setting:** Contrasting with terrestrial concrete that mandates water and extended curing durations, Martian concrete offers rapid setting—a significant advantage in Mars' volatile environment [100].
• **Reusability:** The potential to melt and reshape this concrete implies infrastructural adaptability on Mars [102].

##### Potential limitations and challenges

• **Toxicity:** Extended exposure to certain sulfur compounds could pose health risks. As a precaution, Martian habitats might necessitate interior coatings or barriers to guarantee human safety [103].
• **Thermal Conductivity:** Sulfur concrete's distinct thermal characteristics might influence the insulation capacities of Martian edifices constructed with it [100].
• **Tensile Strength:** Analogous to numerous concretes, Martian variants might possess compromised tensile strength, suggesting potential reinforcements for specific applications [102].

Martian concrete emerges as a frontrunner for realizing robust habitats and infrastructure on the Red Planet. As we refine our understanding and techniques, this material's prominence in Martian construction endeavors is poised to grow. Exploring other construction materials and techniques, such as 3D-printed structures using regolith, also holds great potential [37]. Using Martian regolith for concrete aligns with sustainable practices, offering a feasible solution for long-term habitation. Furthermore, research into enhancing the mechanical properties of Martian concrete, including tensile strength and flexibility, is vital for developing versatile and durable structures [102]. The architectural design of Martian habitats is another critical aspect to consider. Efficient designs must maximize interior space, ensure radiation protection, and accommodate the unique challenges of the Martian environment. Concepts such as modular habitats with expandable sections, buried or semi-buried structures for added radiation protection, and utilization of transparent materials for natural lighting are being explored [6], [96]. Integrating life support systems, energy generation, and waste recycling within habitat designs is also crucial for creating self-sustaining living environments on Mars [88]. Developing Martian infrastructure and habitats is a complex task requiring multidisciplinary collaboration and innovation. From material science to architectural design and engineering, each aspect plays a vital role in transforming the dream of Martian colonization into a reality. The ongoing advancements in these fields continue to bring us closer to establishing sustainable and livable habitats on Mars, paving the way for future human exploration and potential settlement of the Red Planet.

#### 4.1.2 Underground cities

Beyond surface-level habitats, the very substratum of Mars may offer a tantalizing solution to some of the most pressing colonization challenges. Underground cities, or subterranean habitats, can leverage the Martian crust's innate properties to provide shelter, security, and sustainability [89].

##### Natural radiation shielding

• **Protection Depth:** Mars lacks a strong magnetosphere and thick atmosphere, both of which provide Earth with considerable protection from cosmic and solar radiation. By constructing habitats several meters below the surface, colonists can use the regolith and rock as a natural shield, significantly reducing radiation exposure [104].
• **Consistency:** Unlike makeshift shields that may require maintenance and periodic replacement, the consistent composition of the Martian crust provides enduring protection [104].

##### Thermal insulation

• **Stability:** The Martian surface experiences drastic temperature fluctuations. However, subterranean environments tend to maintain a more stable temperature, reducing the energy required for heating and cooling [105].
• **Heat Retention:** The ground naturally serves as an insulator, helping to trap heat and stabilize interior temperatures, especially during prolonged cold periods [105].

##### Resource extraction

• **Water Ice:** Recent discoveries indicate substantial deposits of water ice beneath Mars' surface. Underground habitats can directly access these reserves, providing vital resources for drinking, agriculture, and potentially fuel production [106].
• **Minerals and Metals:** As we dig deeper into Mars, there is potential to discover and extract valuable minerals and metals that can be utilized for in-situ manufacturing and construction [107].

##### Challenges of subterranean living

• **Excavation:** The act of digging and tunneling on Mars poses significant challenges. Martian regolith's properties, combined with the planet's reduced gravity, necessitate the development of new excavation technologies [101].
• **Air Circulation:** Ensuring a consistent and fresh supply of breathable air within underground habitats demands advanced ventilation systems [89].
• **Psychological Aspects:** Extended periods underground could impact colonists' mental well-being. Solutions might include virtual windows, communal gathering areas with simulated natural light, and regular surface excursions [108].

In essence, while underground cities present novel solutions to many Martian challenges, their realization demands significant advancements in excavation technology, habitat design, and psychological support structures [13], [32], [109]. Yet, as our understanding of Mars deepens, so too does the potential for these subterranean havens to play a pivotal role in human colonization [1], [110].

##### Design considerations for subterranean habitats

• **Structural Integrity:** The design of underground habitats must consider the Martian soil's structural integrity and load-bearing capacities. Innovations in architectural design, such as geodesic dome structures and archways, can offer robust solutions [109].
• **Emergency Access and Egress:** Ensuring safe and efficient means for accessing and exiting these underground structures is crucial. This includes redundant airlocks, emergency escape routes, and potentially lift systems for deeper habitats [89].
• **Habitat Expansion:** The modularity and scalability of underground habitats should be factored into initial designs, allowing for future expansion as the colony grows [13].

##### Potential for underground farming and manufacturing

• **Controlled Environment Agriculture:** Underground farming using hydroponic or aeroponic systems can provide a reliable food source. Artificial lighting and climate control systems would be key to supporting plant growth [110].
• **In-Situ Manufacturing:** Utilizing extracted minerals and metals for manufacturing tools, spare parts, and construction materials can significantly enhance self-sufficiency [107].

Underground cities on Mars represent a fusion of innovative engineering, architectural prowess, and adaptive living strategies. These subterranean habitats could provide safe, sustainable, and efficient dwellings for future Martian settlers, leveraging the planet's inherent resources and protecting against its harsh surface conditions.

### 4.2 Energy production

The energy requirements for a Martian colony are multifaceted, requiring a balance between reliability, sustainability, and efficiency [1]. The choice of energy production methods must account for Mars's unique environmental conditions. This subsection explores the various options for generating energy on Mars, assessing their potential and challenges.

#### 4.2.1 Solar energy

While farther from the Sun than Earth, Mars still receives a considerable amount of solar energy. The solar constant on Mars, $I_{mars}$, is approximately half of that on Earth, as given by Eq. (2) [111]:

(2) $$I_{mars}\approx 590\;{\text{W/m}}^{2}$$

Given this, the potential power $P_{solar}$ from a solar panel of area *A* with efficiency *η* can be calculated as shown in Eq. (3) [112]:

(3) $$P_{solar}=\eta \times A\times I_{mars}$$

However, due to dust accumulation and reduced daylight hours during Martian winters, the effective efficiency might be reduced by a factor *f*, as indicated in Eq. (4) [113]:

(4) $$P_{effective}=f\times P_{solar}$$

##### Advancements and challenges in solar technology

• **Dust Mitigation:** Technologies such as electrostatic dust cleaners and self-cleaning surfaces are being developed to mitigate the dust problem on solar panels [23].
• **Efficiency Improvements:** Ongoing research aims to enhance the efficiency of solar panels under Martian conditions, focusing on materials that can withstand extreme temperatures and radiation [114].

#### 4.2.2 Nuclear energy

Nuclear power represents a reliable and potent energy source for Martian colonies, especially during periods when solar energy is insufficient. The power $P_{nuclear}$ produced by a nuclear reactor is defined by Eq. (5) [115]:

(5) $$P_{nuclear}=\eta_{n}\times m\times E$$

where $\eta_{n}$ is the reactor's efficiency, *m* is the mass of the fuel, and *E* is the energy density of the fuel. High energy density and efficient reactors are preferred due to constraints in transporting nuclear fuel from Earth [116].

##### Nuclear reactor designs for Mars

• **Kilopower Reactors:** Compact, lightweight nuclear reactors like Kilopower can provide up to 10 kW of electrical power, potentially scalable for larger power needs [117].
• **Safety and Waste Management:** Advanced designs focus on safety features and efficient waste management, essential for ensuring long-term operational integrity and environmental protection [118].

#### 4.2.3 Wind energy

Despite Mars' thin atmosphere, wind energy remains a potential supplementary power source. The power $P_{wind}$ from wind turbines is calculated using Eq. (6) [65]:

(6) $$P_{wind}=\frac{1}{2}\times \rho \times A\times v^{3}\times \eta_{w}$$

where *ρ* is the atmospheric density of Mars, *A* is the area swept by the turbine blades, *v* is the wind velocity, and $\eta_{w}$ is the efficiency of the wind turbine. The thin atmosphere of Mars reduces the potential efficiency of wind power [119].

##### Innovations in Martian wind turbines

• **Design Optimization:** Turbine designs for Mars focus on large blade areas and materials optimized for low atmospheric density [88].
• **Deployment and Maintenance:** Research includes autonomous deployment, self-repair mechanisms, and dust resistance for long-term operation [120].

#### 4.2.4 Geothermal energy

Exploring the potential of geothermal energy on Mars involves tapping into the planet's internal heat. The expected geothermal power $P_{geo}$ is based on Martian heat flux, as outlined in Eq. (7) [121]:

(7) $$P_{geo}=\eta_{g}\times A_{g}\times q$$

where $\eta_{g}$ is the geothermal plant's efficiency, $A_{g}$ is the area of heat extraction, and *q* is the Martian heat flux. The effectiveness of geothermal energy depends on accurate measurements of Mars' internal heat and advanced drilling technologies [122].

##### Challenges and opportunities in geothermal exploration

• **Heat Flux Measurements:** In-depth studies and measurements of Martian geothermal gradients are required to assess geothermal plants' feasibility and potential locations [123].
• **Drilling Technologies:** Developing robust, autonomous drilling systems capable of penetrating the Martian crust is critical for geothermal energy extraction [101].

Each energy source presents unique advantages and challenges in the Martian context. A combination of these energy technologies, tailored to specific environmental conditions and mission needs, will likely be necessary to ensure a reliable and sustainable energy supply for Martian colonies [1].

### 4.3 Transportation

One of the most formidable challenges in Mars colonization lies in the realm of transportation. Efficient, reliable, and scalable transportation infrastructure is critical to sustain a growing Martian colony. Innovative methods like solar sail-powered cargo ships and advanced rover designs are key to overcoming these logistical hurdles.

#### 4.3.1 Solar sail-powered cargo ships

Solar sails present a revolutionary method of interplanetary transportation, harnessing the Sun's radiation pressure for propulsion. This technology is especially promising for long-duration cargo missions to Mars.

##### Basic principle

Solar sails operate by capturing the momentum of photons from the Sun. While individual photons are massless, their momentum can be transferred to a reflective sail, providing continuous thrust. The propulsion force, $F_{sail}$, exerted by solar photons on the sail, is given by Eq. (8):

(8) $$F_{sail}=\frac{2\times I_{solar}\times A_{sail}}{c}$$

This equation highlights the relationship between the sail's area, $A_{sail}$, and the solar irradiance, $I_{solar}$, at the sail's location.

##### Advantages and challenges

Solar sails offer numerous benefits, including propellant-free travel and the ability for continuous acceleration. However, their practical deployment faces challenges such as efficient initial acceleration, ultra-lightweight yet highly reflective materials manufacturing, and precise navigation control.

##### Applications for Mars

For Mars missions, solar sails could be employed for cargo transport, reducing the dependency on chemical propellants. They could also play a crucial role in establishing a Martian communication network, providing stable, solar-powered platforms for data relay.

##### Emerging concepts and future directions

As material technology and space engineering continue to advance, solar sails might evolve to incorporate features like photovoltaic panels for power generation or be used for more ambitious missions like asteroid mining, further supporting Mars colonization efforts.

#### 4.3.2 Rovers and vehicles for Martian terrain

Rovers and surface vehicles are essential for exploration and logistical support on Mars. Their design must account for the planet's unique terrain and environmental conditions. Considering the Martian challenges, the following design principles are paramount [124], [125]:

• **Robust Mobility:** The rover should feature a multi-wheeled design, often six wheels, to distribute weight and ensure continued mobility even if one wheel malfunctions. The equation governing the force $F_{traction}$ due to Martian gravity is Eq. (9):
  (9) $$F_{traction}=\mu \times m\times g_{mars}$$
  where *μ* is the coefficient of friction, *m* is the mass of the rover, and $g_{mars}\approx 3.71\;{\text{m/s}}^{2}$ is the acceleration due to gravity on Mars.
• **Adaptable Suspension:** To navigate rocky terrains, rovers should have a suspension system that can adjust to irregularities, ensuring all wheels maintain contact with the ground.
• **Dust Mitigation:** To counter the abrasive Martian dust, rovers need protective layers on sensitive parts and possibly electrostatic or mechanical dust removal systems.
• **Thermal Regulation:** Incorporating heaters and insulators to protect against cold and radiative surfaces to dissipate excessive heat is essential.

##### Innovations in rover design

Mars rovers must exhibit robust mobility, adaptability to diverse terrains, and resilience against environmental factors like dust and temperature extremes. For instance, advancements in wheel design, such as shape-shifting wheels or self-cleaning systems, could significantly enhance rover performance on Martian terrain.

##### Manned vehicles for exploration and transport

For manned missions, versatile and robust vehicles are necessary. These might include modular designs for different mission profiles, advanced life support systems, and high-efficiency propulsion systems suited for Mars' lower gravity and thin atmosphere.

##### Integration of autonomous systems

Autonomous navigation and operation capabilities are crucial for both rovers and manned vehicles. Utilizing AI and machine learning for real-time terrain analysis and decision-making could significantly enhance operational efficiency and safety.

##### Challenges in vehicle design and operation

Key challenges include ensuring reliable operation in Mars' harsh environment, developing long-lasting power systems, and addressing the logistical complexities of transporting large vehicle components from Earth to Mars.

The development and refinement of transportation technologies for Mars colonization are a cornerstone of the mission's overall success. Each component, from innovative solar sails to advanced rovers and vehicles, represents a significant step toward establishing a human presence on Mars. These technologies must be functionally effective, economically viable, and sustainable for long-term operations on the Red Planet.

### 4.4 Life support systems

Sustaining human life on Mars presents a series of complex challenges, requiring robust life support systems to meet the basic necessities of air, water, and food. These systems must be designed to function efficiently in the harsh Martian environment, emphasizing sustainability and reliability.

#### 4.4.1 Water harvesting

##### Critical importance of water

Water's role in supporting life on Mars extends beyond basic consumption. It is vital for agriculture, hygiene, and could even be used to produce oxygen and fuel. Efficient water harvesting and management are, therefore, pivotal for the long-term sustainability of Martian colonies.

##### Mars' water resources

Mars possesses diverse water resources, though accessing these reserves presents unique challenges. Subsurface ice, atmospheric moisture, and salt hydrates each offer potential sources but require distinct extraction and processing techniques.

##### Innovative extraction techniques

• **Subsurface Ice Mining:** Extracting water from underground ice reserves involves drilling or digging followed by a heating process to convert ice to vapor [88], [126]. The energy requirement $E_{extraction}$ for this conversion, considering the latent heat of fusion and vaporization, is given by Eq. (10):
  (10) $$E_{extraction}=m_{ice}\times (L_{fusion}+L_{vaporization})$$
• **Atmospheric Water Extractors:** Devices like adsorption-based harvesters can capture atmospheric water vapor during colder periods and release it during warmer times by heating the adsorbent [127].
• **Salt Hydrate Mining:** Extracting water from salt hydrates would involve heating the salts to release the trapped water molecules. This is more energy-intensive and might be viable as a supplementary method [128].

##### Energy requirements and environmental considerations

Each water extraction method has its own energy requirements and potential ecological impacts on Mars. Striking a balance between these factors is crucial. Innovations in renewable energy sources and low-impact extraction methods are being explored to minimize the carbon footprint of these activities.

##### Future prospects

As technology advances, the efficiency and feasibility of these water harvesting methods are expected to improve, significantly enhancing Mars' habitability. The development of autonomous, AI-driven systems for water extraction and management is also on the horizon, potentially revolutionizing how we utilize Martian water resources.

By addressing the challenges of water availability on Mars through innovative technologies and sustainable practices, we can significantly increase the viability of long-term human habitation on the Red Planet. These advancements in life support systems not only pave the way for future Mars missions but also have the potential to offer insights into solving water scarcity issues on Earth.

#### 4.4.2 Aeroponics and food production

##### The need for sustainable agriculture on Mars

Sustainable agriculture on Mars is critical for long-term colonization. The unique Martian environment, lacking organic soil and potential chemical hazards like perchlorates, poses significant challenges for traditional farming methods [88]. Consequently, innovative and resource-efficient agricultural techniques, such as aeroponics, are essential for producing food on Mars [25].

##### What is aeroponics?

Aeroponics, an advanced form of hydroponics, involves growing plants in an air or mist environment without using soil [129]. This method sprays nutrient-rich water directly onto the roots, providing an efficient delivery system for essential minerals and oxygen.

##### Advantages of aeroponics in Martian context

• **Water and Nutrient Efficiency:** Aeroponics can use up to 90% less water than traditional farming, crucial in a water-scarce environment [130]. The water usage *W* for a plant in aeroponics is often given by Eq. (11):
  (11) $$W=V_{spray}\times f_{cycle}\times t_{growth}$$
• **Rapid Plant Growth:** The direct delivery of nutrients and oxygen to plant roots in aeroponics accelerates growth, a valuable trait for ensuring a steady food supply [129].
• **Space Optimization:** Vertical farming techniques in aeroponics make efficient use of limited space in Martian habitats, allowing for a higher yield per square meter [131].
• **Soil Isolation:** By eliminating the need for soil, aeroponics avoids the potential contaminants found in Martian regolith, ensuring safer crop production [88].

##### Challenges and considerations

Despite its advantages, aeroponics in a Martian environment faces several challenges:

• **System Reliability:** Dependence on technology for plant growth necessitates robust and redundant systems to prevent catastrophic failures that could lead to food shortages [132].
• **Nutrient Solution Management:** Precise control over nutrient composition and pH levels is essential for optimal plant growth. This requires advanced monitoring and adjustment systems [129].
• **Adaptation to Martian Gravity:** The impact of Mars' reduced gravity on plant growth and aeroponic systems is not fully understood, necessitating further research and potential modifications to current technology [133].

##### Research and development efforts

To address these challenges, ongoing research focuses on developing more resilient aeroponic systems, optimizing nutrient solutions, and studying plant behavior in simulated Martian gravity conditions. This includes:

• **Automated Monitoring Systems:** Advanced sensors and AI-driven algorithms are being developed to constantly monitor and adjust the aeroponic environment, ensuring optimal growth conditions.
• **Gravity Adaptation Studies:** Experiments in simulated Martian gravity, such as those conducted on the International Space Station, provide insights into how reduced gravity affects plant growth and aeroponic system functioning.
• **Genetic Engineering:** Research into genetically modified crops that are more suited to the unique conditions of Mars, such as enhanced stress tolerance and faster growth rates, is underway.

Integrating advanced aeroponics into Martian colonization strategies represents a significant step toward self-sufficiency. As our understanding and technology continue to evolve, it is likely that aeroponics will play a central role in sustaining human life on Mars, offering a model for sustainable agriculture both on and beyond Earth [134].

#### 4.4.3 Algae bioreactors

##### The role of algae in space colonization

Algae, with their incredible versatility and efficiency in converting light and carbon dioxide into biomass, present a promising solution for several critical functions in space colonization [135]. On Mars, algae could be instrumental in creating a self-sustaining ecosystem, offering solutions for air revitalization, waste recycling, and food supply [136].

##### Benefits of algae bioreactors in a Martian environment

Algae bioreactors, cultivating these organisms under controlled Martian conditions, offer several key benefits [137]:

• **Oxygen Production:** Through photosynthesis, algae convert carbon dioxide into oxygen, a critical process for maintaining breathable air for astronauts. The efficiency of this process is influenced by several factors, including light intensity, the algae species, and the bioreactor design [138]. This process can be modeled as Eq. (12):
  (12) $$6{\text{CO}}_{2}+6{\text{H}}_{2}\text{O}\overset{\text{light}}{\to }{\text{C}}_{6}{\text{H}}_{12}{\text{O}}_{6}+6{\text{O}}_{2}$$
• **Wastewater Treatment:** Algae can absorb and metabolize waste products, including nitrogen and phosphorus, from wastewater, essentially purifying it and making the water recyclable for various uses [139].
• **Nutritional Supplement:** Algae such as Spirulina is rich in proteins, vitamins, and essential amino acids, providing a sustainable food supplement for astronauts [140].
• **Potential for Biofuel:** Certain species of algae can produce lipids, which can be processed into biofuels, a potential renewable energy source on Mars [141].

##### Designing bioreactors for Martian conditions

The design and implementation of algae bioreactors on Mars must consider the planet's unique environmental challenges:

• **Temperature Control:** To account for Mars' temperature extremes, bioreactors need effective insulation and temperature regulation systems to maintain optimal algae growth conditions [142].
• **Radiation Protection:** Mars' thin atmosphere offers limited protection from solar and cosmic radiation, necessitating radiation shielding for bioreactors to prevent DNA damage to the algae [143].
• **Light Management:** With reduced sunlight and possible dust storms, artificial lighting, such as LED systems, could provide consistent and optimal light wavelengths for photosynthesis [144].

##### Research directions and innovations

Ongoing research and development are focusing on enhancing the efficiency and resilience of algae bioreactors for space applications:

• **Genetic Engineering:** Developing genetically modified algae strains that are more resilient to Martian environmental conditions and potentially more efficient in producing oxygen and biomass [143].
• **Closed-Loop Integration:** Integrating algae bioreactors into the habitat's life support system to create a closed-loop system, where waste products are recycled and used by the algae, and the astronauts utilize the oxygen and food produced by the algae [145].
• **Scalable Designs:** Developing modular and scalable bioreactor designs that can be expanded or adapted as the colony grows or as different strains of algae are utilized [135].

In conclusion, algae bioreactors hold immense potential for supporting life on Mars. They could be central to creating a sustainable and efficient closed-loop life support system, contributing significantly to the feasibility of long-term human presence on the Red Planet.

#### 4.4.4 Magnetic shield deployment

##### The radiation threat on Mars

The Martian surface is significantly more exposed to cosmic and solar radiation than Earth due to its lack of a protective global magnetic field and a thin atmosphere [142], [143]. This elevated radiation exposure poses serious risks to human health, potentially increasing the likelihood of cancer, tissue damage, and other detrimental long-term effects.

##### Magnetic shielding: a potential solution

Magnetic shields, designed to create a protective magnetic field, offer a promising approach to mitigate radiation exposure on Mars. These shields would operate similarly to Earth's magnetosphere, deflecting charged particles away from inhabited areas, thereby reducing radiation exposure [142], [143].

##### Working principle

The basis for magnetic shielding is the Lorentz force law, described in Eq. (13):

(13) F=q(v×B)

where **F** is the force experienced by a charged particle, *q* is its charge, **v** is its velocity, and **B** is the magnetic field. In the context of a magnetic shield, this would result in charged particles being deflected, thus preventing them from reaching the Martian surface where they could pose a threat [142].

##### Implementation challenges

Implementing effective magnetic shielding on Mars presents several technical challenges:

• **Power Requirements:** Creating a strong and extensive magnetic field necessitates significant energy. The power required, *P*, can be related to the magnetic field strength, *B*, and the volume of the protected area, *V*, as shown in Eq. (14):
  (14) $$P\propto \eta B^{2}V$$
  where *η* represents efficiency and proportionality factors. This highlights the need for efficient energy sources and advanced power management [144].
• **Magnetic Field Configuration:** Designing an effective magnetic field configuration is complex. Non-uniform fields, such as toroidal or helical shapes, might be required for optimal protection [143].
• **Infrastructure and Weight Constraints:** Developing the necessary infrastructure to generate and maintain these magnetic fields is challenging, particularly considering the limitations in transportation and deployment capabilities in Martian conditions [143].

Despite these challenges, the development of magnetic shielding technology is advancing. Ongoing research in superconductor technology, energy storage, and magnetic field generation is gradually addressing these issues. Additionally, new materials and design concepts are being explored to optimize the efficiency and feasibility of these systems [143], [145]. Magnetic shielding technology holds great promise for enabling safer human habitation on Mars. Providing effective protection against space radiation could significantly reduce one of the major barriers to long-term human presence on the Red Planet. The successful implementation of magnetic shields will depend on a combination of advanced materials, innovative engineering, and sustainable energy solutions [142], [145]. As this technology matures, it could become a key component of the Martian colonies' infrastructure, ensuring future space explorers' safety and health.

### 4.5 Communication: Mars-Earth relay systems

Establishing and maintaining a consistent and reliable communication link between Mars and Earth is crucial for Martian colonies' operational success and safety. The significant distance between the two planets and potential obstructions such as the Sun pose unique challenges. Advanced relay systems, particularly those leveraging the strategic positioning of Lagrange points, offer viable solutions for overcoming these challenges [139], [141].

#### 4.5.1 The Mars L2 Lagrange point

Lagrange points, named after the Italian-French mathematician Joseph-Louis Lagrange, are unique positions in space where the gravitational forces of two large bodies, such as the Sun and Mars, balance the orbital motion of a third, smaller body [146]. Positioning a satellite at one of these points, particularly the Mars L2 point, offers distinct advantages for communication. The effective gravitational potential *V* at this point is given by Eq. (15):

(15) $$V=\frac{1}{2}\omega^{2}r^{2}+\frac{GM_{sun}}{r}+\frac{GM_{mars}}{\left|r-R\right|}$$

Where:

• *ω* represents Mars' angular speed around the Sun.
• *r* is the distance from the Sun to the satellite.
• *R* is the distance from the Sun to Mars.
• *G* is the universal gravitational constant.
• $M_{sun}$ and $M_{mars}$ are the masses of the Sun and Mars, respectively.

For the L2 point to be viable, the effective potential *V* should ideally have a local maximum, offering a relatively stable position for a satellite [147].

#### 4.5.2 Advantages of using the Mars L2 point

Positioning satellites at the Mars L2 point offers several strategic advantages:

• **Constant Line of Sight:** Satellites at this point maintain a continuous line of sight to both Mars and Earth, barring the infrequent alignments when Mars is directly between the Sun and Earth. This unobstructed view ensures consistent communication capabilities [148].
• **Reduced Communication Latency:** A dedicated relay point such as the Mars L2 can facilitate more efficient communication, reducing the time delay inherent in interplanetary communications.
• **Scalability:** The Mars L2 point can support multiple satellites, creating a robust network that enhances redundancy and overall bandwidth capacity, crucial for both operational data and personal communications.

#### 4.5.3 Implementation challenges

Besides the mentioned challenges, placing a satellite at L2 requires careful calculating of its required velocity to maintain its position at the Lagrange point. The required centripetal force $F_{c}$ for the satellite to orbit the Sun in sync with Mars is Eq. (16):

(16) $$F_{c}=m\omega^{2}(R+\delta )$$

Where:

• *m* is the mass of the satellite.
• *δ* is the distance from Mars to the L2 point.

This centripetal force should be balanced by the net gravitational force from the Sun and Mars at the L2 point. Challenges include:

• **Orbital Stability:** While the Mars L2 point is a point of equilibrium, it is not entirely stable. Satellites would require periodic propulsion-based adjustments to remain in position [149].
• **Distance from Mars:** The L2 point is considerably farther from Mars than a typical Martian orbit, meaning satellites placed here would need powerful transmission systems to relay signals effectively to and from the Martian surface.
• **Protection:** Satellites in this position are exposed to higher solar radiation levels, requiring enhanced protective measures to ensure longevity.

Utilizing the Mars L2 point for communication also opens up avenues for scientific observations and studies. Satellites placed here can perform continuous solar observations, monitor space weather, and serve as early warning systems for solar events that could impact Mars and its orbiting spacecraft. As technology evolves, strategies such as deploying solar sails for station-keeping, advanced radiation-hardened electronics, and higher bandwidth communication systems are being researched to make the Mars L2 relay system more effective and reliable. Integration of artificial intelligence for autonomous satellite operation and real-time data processing could further enhance the efficiency of this communication network [150]. The Mars L2 Lagrange point presents a promising solution for addressing the communication challenges between Mars and Earth. Its strategic location, coupled with advancements in space technology, has the potential to establish a robust and reliable communication relay system, which is vital for the success of future Martian exploration and colonization efforts.

### 4.6 Space and surface mobility

The advancement of space and surface mobility technologies is vital for the successful exploration and colonization of Mars [151]. These technologies must be designed to withstand the Martian environment's unique challenges while prioritizing safety, efficiency, and adaptability. This subsection explores the recent progress and future prospects in developing spacesuits, rovers, drones, and other mobility solutions for Mars.

#### 4.6.1 Advanced spacesuits

**Design Requirements:** Spacesuits for Mars missions must meet several essential requirements to protect astronauts in the Martian environment:

• **Radiation Protection:** Due to Mars' thin atmosphere, spacesuits must provide adequate shielding from harmful cosmic and solar radiation [23], [24].
• **Thermal Regulation:** The extreme temperature variations on Mars necessitate spacesuits that can effectively insulate and regulate an astronaut's body temperature [152], [153].
• **Dust Protection:** The omnipresent Martian dust poses a significant threat to both astronauts' health and the integrity of the suit's systems [154].

**Technological Advancements:** Innovative technologies are being developed to enhance the functionality and safety of Martian spacesuits:

• **Layered Shielding:** The use of advanced composite materials with varied properties allows for enhanced radiation protection, as demonstrated in Eq. (17) [155], [156].
  (17) $$E=\int_{0}^{d}\mu (x)\;dx$$
• **Regenerative Cooling Systems:** Implementing phase change materials and endothermic reactions enables the spacesuit to maintain a comfortable temperature in varying environmental conditions [152].
• **Electrostatic Dust Repellents:** Spacesuits may include materials capable of generating a static charge to repel fine Martian dust particles [157].

#### 4.6.2 Rovers and surface vehicles

**Design Considerations:** Rovers and surface vehicles for Mars exploration must be designed with specific considerations in mind:

• **Energy Efficiency:** The extended duration and remote nature of missions necessitate vehicles that maximize energy efficiency [50], [57].
• **Traction and Stability:** The varied Martian terrain requires vehicles capable of maintaining traction and stability over dunes, rocks, and ravines [158].

**Technological Innovations:** Significant technological innovations have been developed to enhance the capability of Martian rovers and vehicles:

• **Hybrid Propulsion Systems:** Integrating solar and nuclear power sources ensures a consistent and reliable energy supply [1].
• **Adaptive Wheel Technology:** Advanced wheel designs that can adjust shape or stiffness improve navigation across varying terrains and extend vehicle lifespan [159].
• **Autonomous Navigation:** Incorporating machine learning algorithms to allow rovers to adapt to new terrains and obstacles without constant input from mission control [31]. The pathfinding algorithm, *P*, can optimize routes based on terrain difficulty, *T*, and energy requirements, *E* (Eq. (18)):
  (18) P=min⁡(αT+βE)
  where *α* and *β* are weighting coefficients.

#### 4.6.3 Other mobility solutions

Other mobility solutions being explored include:

• **Portable Habitats:** Deployable habitats offer flexibility during extended missions, featuring a combination of inflatable and rigid components for balance between portability and protection [160], [161].
• **Drones:** Martian drones, equipped with solar panels, can perform reconnaissance, mapping, and even transport small payloads [162]. These drones must be designed to operate efficiently in the thin Martian atmosphere [163].

**Future Directions:** As technology continues to evolve, future mobility solutions on Mars could include:

• **Advanced Propulsion:** Development of more efficient propulsion systems for rovers and drones to extend their range and capabilities.
• **AI-Driven Navigation:** Enhanced artificial intelligence for autonomous decision-making in complex and unforeseen environmental conditions.
• **Modular and Scalable Design:** Vehicles and habitats with modular components for easy repair, upgrade, and scalability to support growing colonies.

The progress and evolution of space and surface mobility technologies are crucial for successfully exploring and potentially colonizing Mars. Integrating advanced materials, innovative energy solutions, and artificial intelligence will pave the way for more effective, efficient, and safe operations on the Martian surface [164], [165].

## 5 Human factors

Human factors in Martian colonization encompass the complex interplay between humans and the challenging environments they inhabit. The psychological, physical, and social aspects of living on Mars present unique challenges, demanding innovative solutions to ensure the health, well-being, and productivity of Martian settlers [26], [27].

### 5.1 Psychological challenges of isolation and potential solutions

Life on Mars, with its inherent isolation, confinement, and distance from Earth, poses profound psychological challenges [166]. If not adequately addressed, these challenges can affect every aspect of a mission, from individual health to team dynamics and mission success [167].

#### 5.1.1 Sources of isolation and confinement stressors

The sources of psychological stress in Martian habitats include:

• **Distance from Earth:** The vast physical distance from Earth, coupled with communication delays, engenders a profound sense of isolation [166].
• **Limited Social Interactions:** The small number of individuals in Martian habitats and the lack of new social interactions can lead to feelings of monotony and heightened interpersonal tensions [168].
• **Harsh External Environment:** The Martian environment, characterized by its barren landscape and the necessity of wearing spacesuits outdoors, exacerbates the sense of confinement and isolation [169].

#### 5.1.2 Potential psychological impacts

The psychological impacts of these stressors are diverse and can manifest in various ways, including:

• **Mood and Morale:** Prolonged isolation and confinement can lead to decreased mood and morale among crew members [170], [171].
• **Cognitive Performance:** Stress and altered sleep patterns can reduce cognitive performance, impacting mission-critical tasks [171].
• **Sleep Disturbances:** The unusual environment and stress can lead to disrupted sleep patterns, affecting health and performance [170].
• **Interpersonal Conflicts:** Close quarters and limited social outlets may lead to increased interpersonal conflicts and breakdowns in team cohesion [170], [171].

#### 5.1.3 Proposed solutions

Addressing these psychological challenges requires a multi-faceted approach:

• **Advanced Training:** Providing astronauts with comprehensive training in resilience, stress management, and conflict resolution can prepare them for the psychological rigors of Martian living [170].
• **Virtual Reality (VR) Environments:** Utilizing VR technologies to simulate diverse and familiar Earth environments can offer mental escapism and relaxation [171].
• **Telemedicine and Psychological Support:** Ensuring continuous access to psychological support services from Earth, including regular consultations and emergency interventions, is crucial [170].
• **Habitat Design:** Designing habitats with provisions for privacy, communal areas, and green spaces can significantly improve living conditions and mental health.
• **Engaging Activities:** Organizing structured recreational, educational, and creative activities can help combat monotony and maintain high morale.
• **Communication Technology:** Developing technologies that minimize communication delays and facilitate more natural interactions with people on Earth can help reduce feelings of isolation.

In summary, while the challenges of isolation and confinement on Mars are significant, they can be effectively mitigated through a combination of training, technological solutions, and innovative approaches to habitat design and crew activities [170], [171]. Prioritizing the psychological well-being of astronauts is essential for the long-term success and sustainability of Martian colonization efforts.

### 5.2 Training for self-sufficiency given the communication delay with Earth

The substantial communication delay between Mars and Earth, which can vary from 4 to 24 minutes one way, presents a critical challenge for Martian colonists [158]. This delay, combined with the possibility of communication blackouts, necessitates high self-sufficiency for the colony's safety, productivity, and overall success.

#### 5.2.1 Challenges posed by communication delays

Communication delays with Earth pose several unique challenges:

• **Emergency Response:** In emergency situations, quick decision-making is essential, and the delay in communication with Earth eliminates the possibility of immediate guidance or support.
• **Routine Operations:** Daily activities and operations, particularly those involving complex machinery or delicate procedures, are impeded by the inability to receive prompt instructions or feedback from Earth [158].
• **Medical Situations:** In the case of medical emergencies or health issues, the delay can hinder timely medical advice or intervention, necessitating a higher level of medical preparedness and autonomy among the crew.

#### 5.2.2 Training approaches for self-sufficiency

Effective training strategies are critical for preparing astronauts for the communication constraints of Mars:

• **Scenario-Based Training:** Astronauts engage in simulations that replicate various challenges, from routine operations to emergency scenarios, fostering decision-making skills without reliance on real-time Earth input. The training's effectiveness, $E_{t}$, can be assessed by evaluating the success rate in simulated scenarios (Eq. (19)).
  (19) $$E_{t}=\frac{\text{number of successful resolutions}}{\text{number of presented challenges}}$$
• **Cross-Training:** To ensure redundancy and versatility in skills, astronauts are cross-trained in various disciplines. Engineers might learn basic medical skills, while medical personnel might be trained in essential engineering tasks.
• **Utilizing AI and Advanced Simulations:** Integrating AI and virtual reality simulations provides astronauts with tools to practice and refine skills, simulating a wide array of scenarios they might encounter on Mars.
• **Continuous Learning Modules:** Astronauts have access to ongoing learning modules regularly updated with the latest research, techniques, and innovations from Earth, ensuring they stay abreast of current knowledge and practices.
• **Behavioral and Psychological Training:** Training also encompasses psychological resilience, conflict resolution, and stress management skills, which are crucial for maintaining team dynamics and mental health in the isolated Martian environment [170].

#### 5.2.3 Feedback and iterative improvement

Iterative improvement through feedback is a core component of the training process. After each training scenario, astronauts engage in debriefings to analyze the outcomes, discuss alternatives, and integrate lessons learned. This feedback loop, represented as *F*, can be quantified by changes in training efficacy across scenarios (Eq. (20)):

(20) $$F=\frac{\Delta E_{t}}{\text{number of training scenarios}}$$

Training for self-sufficiency in the face of Mars-Earth communication delays is a multifaceted challenge, requiring a comprehensive approach encompassing technical, medical, and psychological aspects. By adopting these training methods and emphasizing autonomous problem-solving, Martian colonists can better handle the diverse challenges they will encounter on the Red Planet [158].

### 5.3 Health considerations in reduced gravity

Mars' gravity, at approximately 38% of Earth's, presents significant challenges for human health and physiology [172], [173]. Adapting to this reduced gravity environment can lead to various physiological changes, impacting the health, performance, and long-term well-being of Martian settlers [174]. Understanding and mitigating these effects are paramount for the successful habitation of Mars.

#### 5.3.1 Bone density loss

• **Description:** The lower mechanical load on bones in reduced gravity leads to decreased bone mineral density, potentially resulting in conditions like osteopenia or osteoporosis [175], [176].
• **Quantifying Loss:** The rate of bone density loss, $R_{b}$, is influenced by the gravitational difference and can be approximated using Eq. (21):
  (21) $$R_{b}=-k\times (g_{m}-g_{e})$$
  Where *k* is a proportionality constant, $g_{m}$ is Mars' gravity, and $g_{e}$ is Earth's gravity [177].
• **Mitigation Strategies:** Strategies include regular weight-bearing exercises, pharmacological interventions, and a diet rich in bone-strengthening nutrients like calcium and vitamin D [176], [178].

#### 5.3.2 Muscle atrophy

• **Description:** The reduced muscle load in lower gravity environments leads to muscle weakening or atrophy [175], [179].
• **Quantifying Atrophy:** Muscle atrophy rate, $R_{m}$, can be similarly represented (Eq. (22)):
  (22) $$R_{m}=-k^{\prime }\times (g_{m}-g_{e})$$
  Where $k^{\prime }$ is a proportionality constant [179].
• **Mitigation Strategies:** Effective strategies include regular resistance training, neuromuscular electrical stimulation, and maintaining a balanced diet rich in proteins and essential nutrients [180], [181].

#### 5.3.3 Cardiovascular changes

• **Description:** The reduced gravity environment can lead to cardiovascular adaptations like orthostatic intolerance and changes in blood volume distribution [182], [183].
• **Mitigation Strategies:** Strategies include using compression garments to aid venous return, engaging in regular cardiovascular exercises to maintain heart health, and effective hydration strategies [182], [184].

#### 5.3.4 Visual impairment

• **Description:** Changes in intracranial pressure due to fluid shifts can lead to visual impairments, a condition referred to as spaceflight-associated neuro-ocular syndrome (SANS) [185].
• **Mitigation Strategies:** Regular monitoring of eye health, using therapeutic eyewear or corrective lenses, and adjusting the ambient pressure in living quarters can help mitigate these effects [185], [186].

#### 5.3.5 Immune system alterations

• **Description:** Reduced gravity may also impact the human immune system, leading to changes in immune cell function and increased susceptibility to infections [187].
• **Mitigation Strategies:** Enhancing overall health through nutrition, exercise, stress reduction, and targeted research into immune support supplements or medications [187], [188].

#### 5.3.6 Psychological health

• **Description:** The psychological impacts of living in a reduced gravity environment, combined with isolation and confinement, can include mood disorders, cognitive decline, and social issues [26], [189].
• **Mitigation Strategies:** Implementing comprehensive psychological support systems, providing leisure and recreational activities, and facilitating regular communication with family and friends on Earth [26], [189].

Understanding and addressing the health considerations of reduced gravity is essential for the long-term success of Martian colonization. Through a combination of preventive measures, ongoing research, and adaptive strategies, it is possible to mitigate the adverse effects and ensure the health and functionality of humans living on Mars [172], [173].

## 6 Economic considerations

### 6.1 Cost analysis of using in-situ resources vs. transport from Earth

The economic viability of Martian colonization is critical, influencing the mission's feasibility and long-term sustainability [1], [190]. A key decision is between leveraging in-situ Mars resources versus transporting materials from Earth [191]. This subsection examines the cost-benefit analysis of these approaches, considering factors like transportation costs, technology development, and resource availability [192].

#### 6.1.1 Transport costs from Earth

• **Description:** Launching payloads from Earth to Mars is a significant cost driver. These expenses include the rocket and fuel costs, ground infrastructure, labor, and other associated costs [190], [193].
• **Quantifying Transport Costs:** The cost of transporting a unit mass, $C_{t}$, can be calculated as shown in Eq. (23):
  (23) $$C_{t}=C_{launch}+C_{infra}+C_{labor}+C_{misc}$$
  Where:
  **–** $C_{launch}$ includes the rocket and fuel costs [190].
  **–** $C_{infra}$ covers ground infrastructure expenses.
  **–** $C_{labor}$ encompasses manpower costs.
  **–** $C_{misc}$ comprises other miscellaneous expenses.

#### 6.1.2 In-situ resource utilization (ISRU) costs

• **Description:** Utilizing Martian resources, such as water and regolith, for essentials like fuel, air, and construction materials can reduce Earth transportation needs [29], [194].
• **Quantifying ISRU Costs:** The cost per unit mass produced via ISRU, $C_{isru}$, is detailed in Eq. (24):
  (24) $$C_{isru}=C_{tech}+C_{op}+C_{maint}$$
  Where:
  **–** $C_{tech}$ is the initial cost for setting up ISRU technology on Mars [194].
  **–** $C_{op}$ covers ongoing operational costs, including energy consumption.
  **–** $C_{maint}$ involves maintenance and potential repair expenses.

#### 6.1.3 Extended cost-benefit analysis

The comparison of $C_{t}$ and $C_{isru}$ reveals the direct cost implications of each approach. However, a comprehensive analysis must also consider:

• **Scalability:** As Martian operations scale, the cost-effectiveness of ISRU is likely to improve due to economies of scale, unlike the relatively fixed costs of Earth-based launches [42].
• **Sustainability:** Long-term sustainability and environmental impact on Mars should be factored into economic models, giving an advantage to ISRU, which promotes self-sufficiency [1].
• **Technological Risks:** The potential risks associated with technology failure in transport and ISRU operations must be factored into the cost calculations [195].
• **Economic Opportunities:** The development of ISRU technologies could spur new economic opportunities, such as mining and processing Martian resources for local use or export back to Earth [191].

Incorporating these additional economic dimensions into the analysis will offer a more nuanced understanding of the economic implications of Martian colonization [190], [193]. As ISRU technology matures, its role in making Martian colonization economically viable and sustainable is expected to become increasingly significant [29].

### 6.2 Potential economic incentives for private companies to invest in Mars colonization

The ambition of Mars colonization extends beyond scientific and engineering challenges to encompass significant economic potential. The magnitude and complexity of such an endeavor demand substantial capital, making the role of private-sector investment crucial [1], [190]. This section explores the incentives that could attract private companies to invest in Mars exploration and development.

#### 6.2.1 Extraction and utilization of Martian resources

• **Description:** Mars harbors diverse resources, including water-ice, various metals, and rare minerals, which can be leveraged for Martian development and potential export [196].
• **Economic Model:** The expected profit, $P_{res}$, from resource extraction can be quantified as follows (Eq. (25)):
  (25) $$P_{res}=R_{sell}-(C_{extraction}+C_{transport})$$
  Where:
  **–** $R_{sell}$ represents the revenue from resource sales.
  **–** $C_{extraction}$ includes the costs of extraction on Mars.
  **–** $C_{transport}$ accounts for the costs of transporting resources.

#### 6.2.2 Technological development and intellectual property

• **Description:** The challenges in Mars colonization necessitate the development of new technologies, offering opportunities for patents and intellectual property licensing [197].
• **Economic Model:** The revenue from technological innovation, $R_{tech}$, can be calculated as (Eq. (26)):
  (26) $$R_{tech}=R_{license}+R_{adapt}$$
  Where:
  **–** $R_{license}$ is income from licensing technologies.
  **–** $R_{adapt}$ represents revenue from adapting technologies for Earth or other space applications.

#### 6.2.3 Tourism and real estate development

• **Description:** The prospect of Mars as a destination for tourism and potential real estate developments offers new market opportunities [190], [198].
• **Economic Model:** Projected revenue from tourism, $R_{tourism}$, can be modeled with the following equation (Eq. (27)):
  (27) $$R_{tourism}=N_{visitors}\times (T_{ticket}+T_{stay})$$
  Where:
  **–** $N_{visitors}$ denotes the number of tourists.
  **–** $T_{ticket}$ and $T_{stay}$ are revenues from travel and accommodations, respectively.

#### 6.2.4 Collaborations and public relations

• **Description:** Participation in Mars colonization projects can significantly enhance a company's image, leading to partnerships and brand value enhancement [199].
• **Economic Impact:** These intangible benefits can open doors to lucrative government contracts, research partnerships, and an increased market share through enhanced public perception.

#### 6.2.5 Research and development tax incentives

• **Description:** Governments often offer tax incentives for research and development activities, which can significantly benefit companies investing in Martian technology and exploration [200].
• **Economic Impact:** These incentives can reduce the overall investment cost in Mars-related research and development, making it a more attractive proposition for companies [200].

#### 6.2.6 Supply chain and market expansion

• **Description:** Establishing a supply chain for Mars colonization can lead to the development of new markets, both in space and on Earth, with the potential for significant economic growth [199].
• **Economic Model:** The expansion into Martian markets can be seen as an opportunity to diversify and grow a company's revenue streams.

## 7 Timeline for Mars colonization

### 7.1 Overview

A comprehensive timeline for Mars colonization is essential for understanding the sequence and dependencies of key milestones in this monumental endeavor. This timeline is based on current technological capabilities, projected advancements, and anticipated logistical and financial commitments. It's important to note that this timeline is speculative and subject to change based on future technological innovations, funding priorities, and international collaboration dynamics.

### 7.2 Early phase: robotic exploration and technology demonstration (2020s - 2030s)

• **2020s:** Continued robotic exploration. Missions like NASA's Perseverance and the ESA's ExoMars will focus on astrobiology and sample return. Advancements in autonomous navigation, life support systems, and In-Situ Resource Utilization (ISRU) technologies.
• **Early 2030s:** Test missions for human life-support systems and ISRU on the Martian surface. Possible construction of an uncrewed Mars base.

### 7.3 Manned missions: initial human landing and short-term habitation (2030s - 2040s)

• **Mid-2030s:** First crewed mission to Mars. Short-term stays focused on research, habitat establishment, and ISRU exploitation.
• **Late 2030s - Early 2040s:** Establishment of a permanently inhabited base. The expansion of ISRU capabilities focuses on water extraction, oxygen production, and possibly agriculture.

### 7.4 Expansion phase: developing a sustainable colony (2040s - 2060s)

• **2040s:** Expansion of the base into a self-sustaining colony. Development of agriculture, manufacturing, and increased focus on scientific research.
• **2050s:** Growth in the number of inhabitants. Enhanced transport systems between Earth and Mars. Development of Martian industries and the possible beginning of economic returns from resource utilization.
• **2060s:** Continued expansion and potential for larger-scale colonization. Focus on quality of life, long-term sustainability, and Martian-born human research.

### 7.5 Long-term vision: a thriving Martian society (2070s and beyond)

• **2070s and Beyond:** Mars hosts a self-sustaining society with a diverse economy, culture, and scientific community. Regular travel between Earth and Mars. Mars serves as a hub for further solar system exploration.

### 7.6 Conclusion

This timeline presents a framework for Mars colonization, aligning technological development, human exploration, and settlement phases. It underscores the incremental nature of space exploration, where each phase builds upon the successes and lessons of the previous ones. It's a vision requiring decades of dedicated effort, international collaboration, and sustained financial commitment.

## 8 Recommendations for future research

This study emphasizes the need for continued research in various domains to support the viability and success of Mars colonization. Future research should focus on refining resource utilization techniques, developing sustainable energy solutions, enhancing communication systems, and understanding the long-term impacts of reduced gravity on human health. Additionally, economic models must evolve to capture the dynamics of space exploration and Mars colonization, considering technological advances, policy changes, and market developments. Integrating private-sector capabilities and interests will be crucial in advancing these efforts, potentially leading to groundbreaking discoveries and technological innovations. Mars colonization represents a monumental scientific and engineering challenge and a significant economic opportunity. By harnessing the potential incentives, private companies can contribute to humanity's next great venture into space while realizing substantial economic benefits.

## 9 Conclusion

The pursuit of Mars colonization, an ambition once nestled in the pages of science fiction, is now evolving into a tangible goal within our technological and scientific reach. This paper has thoroughly explored the multifaceted aspects necessary for establishing human habitation on Mars, underlining the importance of a multidisciplinary approach that intertwines environmental, technological, human, and economic considerations. Central to the sustainability of Martian colonization is the strategic use of in-situ resources, a key factor in reducing Earth dependency and revolutionizing interplanetary settlement from both logistical and economic perspectives. This approach opens doors to novel economic opportunities and scientific discoveries, pivotal in making Mars colonization feasible and viable. The human aspect of Mars colonization is equally critical. Ensuring astronauts' physical and psychological well-being necessitates comprehensive strategies encompassing advanced life support systems, psychological support, and extensive training. The challenges of reduced gravity, isolation, and adaptation to a new environment demand robust solutions prioritizing Martian settlers' health and safety. Technological advancements are the backbone of Mars colonization. From developing durable spacesuits and habitats to establishing efficient energy and communication systems, each technological breakthrough brings us closer to realizing the goal of living on Mars. However, these innovations must be specifically tailored to meet Mars's unique challenges and conditions. Economically, the venture into Mars colonization requires a pragmatic approach. Public and private investments should be directed towards initiatives that offer scientific and potential economic returns. The involvement of the private sector and international collaboration is crucial for funding this ambitious endeavor. The journey to Mars symbolizes humanity's enduring spirit of exploration and quest for knowledge. It marks a critical step in our expansion beyond Earth, driven by our desire to understand the universe, stimulate technological and scientific advancement, and inspire future generations. The path is marked with complexities and challenges, yet with collaborative efforts, innovative solutions, and strategic planning, Mars colonization is within our potential reach. This paper has outlined a feasible timeline for this monumental endeavor, from preliminary unmanned missions for resource assessment to short-term manned missions, eventually leading to long-term, sustainable settlements. As we stand on the brink of this new era in human history, our actions and decisions must be guided by caution, creativity, and unwavering commitment to scientific integrity and ethical responsibility. Mars colonization transcends the goal of establishing a human presence on another planet; it represents a journey to broaden our cosmic understanding, catalyze growth in various fields, and ignite people's imaginations worldwide. The complexities and challenges are immense, but with collaborative effort, innovative solutions, and thoughtful planning, the red sands of Mars could well be within our reach, marking a monumental milestone in our cosmic journey.

## Ethics approval

Not applicable.

## Consent to participate

Not applicable.

## Consent for publication

Not applicable.

## Author contributions

Dr. Florian Neukart was the sole contributor to this research work. He conceptualized the study, conducted all the research, performed data analysis, and wrote the entire manuscript.

## Funding

The authors did not receive support from any organization for the submitted work.

## Availability of data and material

No data except other research articles and books were used. These are listed in the References section.

## Code availability

No code was produced for this study.

## CRediT authorship contribution statement

**Florian Neukart:** Writing – review & editing, Writing – original draft, Validation, Supervision, Resources, Project administration, Methodology, Investigation, Conceptualization.

## Declaration of Competing Interest

The authors declare that they have no known competing financial interests or personal relationships that could have appeared to influence the work reported in this paper.

## Data Availability

Data sharing does not apply to this article as no new data were created or analyzed in this study.
